# Exponentially selective molecular sieving through angstrom pores

**DOI:** 10.1038/s41467-021-27347-9

**Published:** 2021-12-09

**Authors:** P. Z. Sun, M. Yagmurcukardes, R. Zhang, W. J. Kuang, M. Lozada-Hidalgo, B. L. Liu, H.-M. Cheng, F. C. Wang, F. M. Peeters, I. V. Grigorieva, A. K. Geim

**Affiliations:** 1 Department of Physics and Astronomy, University of Manchester, Manchester, M13 9PL UK; 2 National Graphene Institute, University of Manchester, Manchester, M13 9PL UK; 3 Department of Physics, University of Antwerp, Groenenborgerlaan 171, B-2020 Antwerp, Belgium; 4 NANOlab Center of Excellence, Groenenborgerlaan 171, B-2020 Antwerp, Belgium; 5 Department of Photonics, Izmir Institute of Technology, 35430 Izmir, Turkey; 6 Graphene Center, Tsinghua-Berkeley Shenzhen Institute, Tsinghua Shenzhen International Graduate School, Tsinghua University, Shenzhen, 518055 China; 7 Graphene Center, Institute of Materials Research, Tsinghua Shenzhen International Graduate School, Tsinghua University, Shenzhen, 518055 China; 8 Chinese Academy of Sciences Key Laboratory of Mechanical Behavior and Design of Materials, Department of Modern Mechanics, University of Science and Technology of China, Hefei, 230027 Anhui China

**Keywords:** Nanoscale materials, Graphene

## Abstract

Two-dimensional crystals with angstrom-scale pores are widely considered as candidates for a next generation of molecular separation technologies aiming to provide extreme, exponentially large selectivity combined with high flow rates. No such pores have been demonstrated experimentally. Here we study gas transport through individual graphene pores created by low intensity exposure to low kV electrons. Helium and hydrogen permeate easily through these pores whereas larger species such as xenon and methane are practically blocked. Permeating gases experience activation barriers that increase quadratically with molecules’ kinetic diameter, and the effective diameter of the created pores is estimated as ∼2 angstroms, about one missing carbon ring. Our work reveals stringent conditions for achieving the long sought-after exponential selectivity using porous two-dimensional membranes and suggests limits on their possible performance.

## Abstract (web summary)

Two-dimensional membranes with angstrom-sized pores are predicted to combine high permeability with exceptional selectivity, but experimental demonstration has been challenging. Here the authors realize angstrom-sized pores in monolayer graphene and demonstrate gas transport with activation barriers increasing quadratically with the molecular kinetic diameter.

## Introduction

Two-dimensional (2D) membranes with a high density of angstrom-scale pores can be made by engineering defects in 2D crystals^1–9^ or, perhaps more realistically in terms of applications, by growing intrinsically porous crystals such as, e.g., graphynes^10–12^. Interest in angstroporous 2D materials is strongly stimulated by potential applications, particularly for gas separation as an alternative to polymeric membranes employed by industry^3,13^. On one hand, the atomic thickness of 2D materials implies a relatively high permeability as compared to traditional 3D membranes. On the other hand, angstrom-scale pores with effective sizes *d*_P_ smaller than the kinetic diameter *d*_K_ of molecules should pose substantial barriers for their translocation, which is predicted to result in colossal selectivities *S* > 10^10^, even for gases with fractionally (∼25%) different *d*_K_ such as, for example, H_2_ and CH_4_^1,14,15^. This unique combination of material properties holds a promise of better selectivity-permeability tradeoffs than those possible by conventional membranes^3,13^. At present, this optimistic assessment is based mostly on theoretical modeling. Experimental clarity has so far been achieved only for the classical regime of *d*_P_ > *d*_K_ where the flow is governed by the Knudsen equation, and the resulting modest selectivities arise from differences in thermal velocities of gases having different molecular masses *m*^7–9,16^. For smaller pores with *d*_P_ ≈ *d*_K_, *S* up to 10–100 have been reported for monolayer graphene^5,8^, and even higher selectivities (∼10^4^) were found for some defects with an estimated diameter of ∼3.5 Å in bilayer graphene^4^. Still, this is many orders of magnitude smaller than *S* predicted for the activated-transport regime, *d*_P_ < *d*_K_^1,14,15^. Little remains known about the latter regime, which has proven to be extremely difficult to reach in experiment^5,8,9^. Indeed, even monovacancies in dichalcogenide monolayers were suggested to exhibit the conventional Knudsen flow^9^. The experimental difficulties and lack of understanding are further exacerbated by prohibitive computational costs of simulating molecular permeation in the activated regime^17–20^.

In this work, we achieve the activated regime by creating individual angstrom-scale pores in monolayer graphene by its short exposure to a low-energy electron beam. Gas permeation measurements reveal exponentially large selectivities with activation barriers that depend quadratically on gas molecules’ kinetic diameter.

## Results

### Experimental devices

**Fig. 1:**
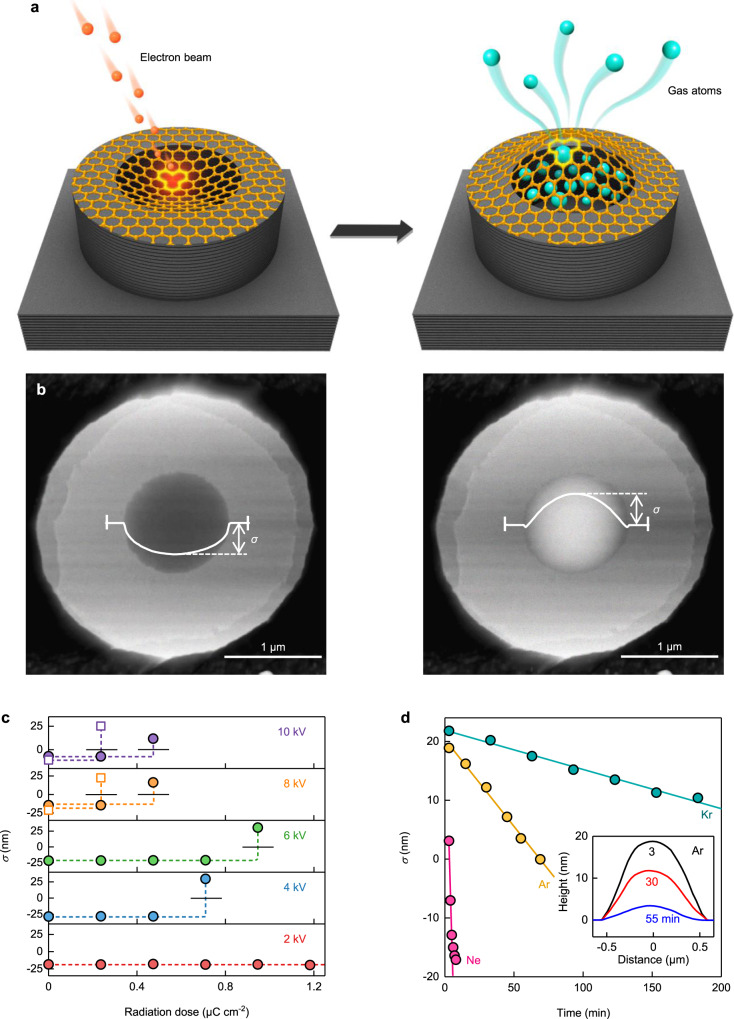
Creating defects in suspended graphene. **a** Schematic of our devices. Left: Monolayer graphene sealing a microcavity was bombarded with electrons. Initially, the membrane sagged inside the cavity due to adhesion to the side walls^4,5,21^. Right: After pressurization, defected membranes bulged out. **b** AFM images of the same device before (left) and after (right) its exposure to 10 keV electrons; dose of 0.5 μC cm^−2^. Both images were taken after storing the device in Kr at 3 bar for 10 days. The white curves are height profiles along the membrane diameter^21^. *σ* is the membrane’s central position measured with respect to graphite’s top surface. The gray scale is given by *σ* ≈ −15 and +24 nm in the left and right images, respectively. **c** Examples of *σ* as a function of radiation dose and acceleration voltage. Each point is taken after pressurizing the devices in 3-bar Kr. Dashed lines: guides to the eye; short black lines: *σ* = 0. **d** *σ*(*t*) for a device with the medium-size pore denoted as type 2, after pressurizing it with various gases (color coded). Solid curves: best linear fits. Inset: representative height profiles for a deflating device with Ar inside.

Our devices were micrometer-size cavities sealed with monolayer graphene (Fig. 1a). The microcavities were fabricated from graphite monocrystals, using lithography and dry etching, and had internal diameters of 1–3 μm and depth of ∼100 nm (“Methods”). Large, exfoliated graphene crystals were then transferred in air on top of the microcavities, creating “atomically tight” sealing^21^. The sealing was tested by placing the devices into a He atmosphere and monitoring changes in graphene membrane’s position by atomic force microscopy (AFM) (Fig. 1b). We selected only the devices that were completely impermeable to He (“Methods”; ref. ^21^). Next, the He-tight membranes were subjected to electron radiation using a scanning electron microscope. The accelerating voltage was chosen to be ≤10 kV, and the beam current was set at 10 pA. In a single exposure lasting 3–5 s and using magnification of 700, an area of ~150 × 150 μm^2^ was radiated, which translated into an electron dose of 0.1–0.2 μC cm^−2^ or only ∼1 electron per 100 nm^2^. After the exposure, the devices were He-leak tested again. The procedure was repeated several times, until a leak appeared indicating a damage induced by electrons (Fig. 1c).

### Number of pores

We argue that, in most cases, only a single pore was created during such exposures. This conclusion is supported by the following observations. First, the pores appeared after extremely small doses of <0.01 electron per typical pore (its size is determined later in the report). Second, the pores appeared suddenly, usually after not one but several such exposures (Fig. 1c), and no additional damage or modification normally occurred during further, much longer exposures (>100 times larger doses; Supplementary Fig. 3). Third, no pores could be created in ~20% of our devices even after hour-long exposures. This clearly shows that the observed perforation was not a continuous damage process but represented rare single events. They can be attributed to the presence of “weak spots” where damage could be induced by the electron beam. Most of our devices had only one such spot, whereas the others had none or two, which would explain the above observations (Supplementary Fig. 3). Fourth, we followed ref. ^5^. and sealed the leaking pores with sparsely dispersed Au nanoparticles (Supplementary Information; Supplementary Fig. 2), an approach used previously to argue the presence of individual pores in graphene membranes. Fifth and most unequivocal, only three discrete pore sizes were ever observed in our experiments rather than their statistical distribution (see below). If several pores were present in a single membrane, a broad distribution of leak rates should have been observed. All this evidence suggests the presence of a single pore in our typical device.

As for a mechanism of creating such pores, incident electrons with an energy of 10 keV can transfer at most ~1.8 eV to a carbon atom, which is ten times less than the threshold energy (18–20 eV) required for knock-on damage^22,23^. Many electrons would be needed to strike the same carbon atom nearly simultaneously to remove it from the graphene lattice, which is statistically impossible especially for the used low doses. Accordingly, we tentatively attribute the pore formation to “chemical etching” of graphene with locally adsorbed water, which was activated by the electron beam, as reported previously^24–26^. We also speculate that further electron-beam exposure protected graphene from continuous water-mediated damage because hydrocarbons adsorbed on graphene became cross-linked^27,28^ and prevented water molecules from reaching the surface. This would be consistent with our observation of rare damage events and the absence of the pores’ modification during further radiation. It would be interesting to gain further information about the discussed etching processes, which can probably be achieved by numerical simulations.

### On impossibility of imaging individual atomic-scale pores in graphene

Unfortunately, no existing technique can visualize the created pores’ atomic structures. Indeed, let us first compare the doses used in our experiments with those typical for studies of graphene defects by high-resolution transmission electron microscope (HRTEM). In the latter case, beam currents were ~10^5^–10^6^ electron nm^−2^ s^−1^ with exposure times of many seconds^22,27,29,30^. In contrast, our pores were created using a dose of only ~10^−2^ electron per square nm, at least seven orders of magnitude lower than needed for HRTEM imaging. Furthermore, we used low-energy (typically 8 kV) beams whereas atomic-resolution TEMs operate at 60 kV or higher. The combination of the high doses and high acceleration voltages required for HRTEM imaging would inevitably result in additional defects in graphene or modification of the existing ones. Even if we were to find a rare angstrom-size pore in our large μm-scale membranes, it would be impossible to argue that the defect was previously there, created by the low-energy beam rather than emerged during the imaging, leaving aside the fact that defects in graphene are known to be strongly modified by > 60 kV used for HRTEM imaging. The fact that it is practically impossible to visualize the studied pores also applies to AFM and scanning tunneling microscopy (STM). Although AFM allows the atomic resolution using freshly cleaved graphite or multilayer graphene, monolayer graphene presents a much harder challenge, especially because of mechanical instabilities induced by the tip interacting with suspended membranes. Vacancies and other atomic-scale defects were previously imaged by STM using atomically clean graphene^31,32^ but our membranes after the electron-beam exposure are not clean or flat, being covered by an atomically thin layer of hydrocarbon contamination^4,5,27^. Not surprisingly, all the previous reports on individual pores in graphene could not visualize them either^4,5^.

### Gas permeation through the atomic-scale pores

The defected membranes prepared as described above were subjected to further permeation tests using various gases (namely, He, Ne, Ar, Kr, Xe, H_2_, CO_2_, O_2_, N_2_ and CH_4_). To this end, the devices were placed in a chamber containing a mixture of air at 1 bar (to match the air captured inside during fabrication) and the tested gas at a partial pressure *P* of typically ≥3 bar. Storage for 2–20 days, depending on the gas, allowed pressures inside and outside to equalize so that the membranes reached stable-in-time positions. After taking the devices back into air, graphene membranes would normally bulge out (Fig. 1a, b) and then gradually deflate, which was monitored by AFM (Fig. 1d). For quantitative analysis, we recorded the central position *σ* of bulged membranes (Fig. 1b) as a function of time *t*. Initially, *σ* evolved linearly with *t*, indicating a constant outflow of the tested gas (Fig. 1d), until its partial pressure inside dropped leading to saturation in *σ*(*t*), in agreement with the behavior reported in refs. ^4,5^. We used the initial slope to evaluate the permeation rate *J* for each gas, as described in Supplementary Information. Repeating this procedure at different *P*, we confirmed that *J* ∝ *P* (Supplementary Fig. 1) and, therefore, the pores could be characterized by their *P*-independent permeance J* = *J*/*P*. For slowly permeating gases, our range of J* was limited by observation times of several days, which yielded a permeance of ∼10^−31^ mol s^−1^ Pa^−1^, that is, less than one gas atom per minute escaping the cavity. It is due to this exceptional sensitivity that we could detect flows through individual pores in the activated-transport regime, which would be difficult if not impossible to access otherwise^4,5,9,21^. As for the upper limit on J*, it was determined by the required time of ∼3 min to obtain an AFM image after taking devices from the gas chamber, which translates into ∼10^−23^ mol s^−1^ Pa^−1^, if using high *P* = 10 bar and our largest cavities.

**Fig. 2:**
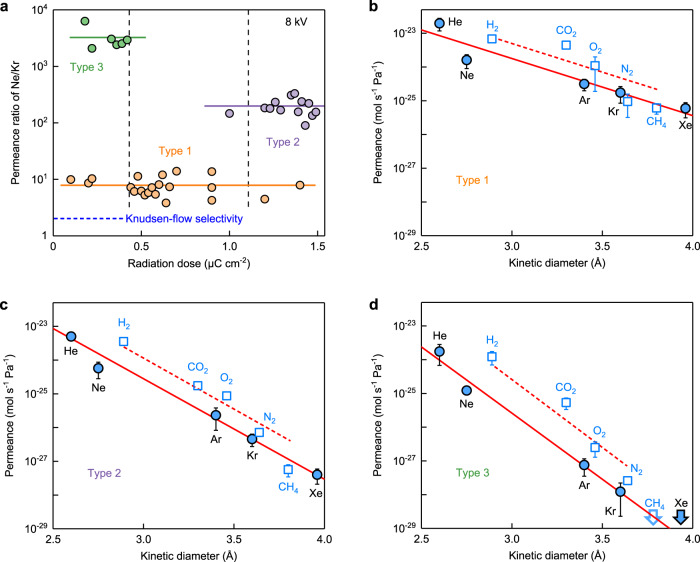
Gas selectivity for graphene pores created by electron bombardment. **a** Selectivity between Ne and Kr as a function of the dosage at which the pores appeared under an 8 kV electron beam. Each symbol denotes a different device. Three distinctive groups are emphasized by their color with the solid lines indicating the average *S* for each group. Vertical lines: guides to the eye indicating typical threshold doses for different pore types. **b**–**d** J* for the three types of pores using ten different gases, as annotated in the panels. Error bars: SD for typically six but minimum three devices. Solid curves in **b**–**d**: best fits to the exponential selectivity J* ∝ exp (−*αd*_K_) for noble gases with *α* being constants. Because of the limited range of *d*_K_, the data fit equally well with J* ∝ exp (−*αd*_K_^2^) (not shown). Dashed curves: guides to the eye for diatomic gases. The arrows in **d** refer to undetectable permeation for Xe and CH_4_.

Our measurements of J* are summarized in Fig. 2 on the basis of more than 40 devices, with each one used to probe several gases. Only three distinct types of pores were observed. This is illustrated by Fig. 2a that compares J* for Ne and Kr (30% different *d*_K_). The measured selectivities *S* = J*(Ne)/ J*(Kr) fall into clearly separated groups. Small scattering around the average *S* within each group can be attributed to random local strain or curvature^21^. We refer to the groups as type 1, 2 and 3 pores, according to their *S*. Using other acceleration voltages between 4 and 10 kV, again only the same three types of pores were observed. This is the strongest evidence in favor of only one pore per membrane (see the other arguments above). The only possibility we cannot rule out is that, for membranes exhibiting highest permeance, two types of pores could be present. For example, type 3 pore could in principle be also present in some devices referred to as type 1 because the biggest pore should dominate the permeation rate. Even if such statistically unlikely events did happen, this would not change any of our conclusions below.

Figure 2a also shows that the radiation dose at which a pore appeared can serve as a good predictor of its type, before doing actual gas permeation measurements, with low and high doses favoring type 3 and 2 pores, respectively. The observed nonmonotonic dependence of pores’ permeability on radiation dose seems surprising. Indeed, the appearance of bigger pores for larger doses as in the case of type 1 and 3 pores is what is generally expected. To obtain tighter pores (type 2) using doses higher than those allowing the largest pores (type 1) is somewhat counterintuitive. Note however that, in all the cases, the pores appeared spontaneously at some weak spots and did not evolve further with increasing the dose (Supplementary Fig. 3). We speculate that the weak spots are determined by local strain and/or random adatoms on the graphene surface and, once such a spot is in place, the low-energy beam would eventually activate its damage into a predetermined structural configuration.

Characteristics of each pore type are detailed in Fig. 2b–d. All the pores exhibited exponential dependences J*(*d*_K_) with type 3, the least permeable pores, being most selective, followed by type 2 and 1. Judging by their permeance, type 1 pores are similar to those created by ultraviolet-induced oxidation^5^. Within our sensitivity limits, the smallest (type 3) pores were completely impermeable to Xe and CH_4_ yielding selectivity > 10^7^ with respect to He or H_2_, which is higher than *S* for any type of membranes reported in the literature. Surprisingly, diatomic gases exhibited systematically higher J* than noble gases (Fig. 2). This cannot be due to the elongated shape of diatomic molecules because *d*_K_ corresponds to the smallest cross-section^33^, that is, the most favorable orientation for translocation. Furthermore, Fig. 2 shows that the observed permeation was controlled mainly by spatial confinement rather than, e.g., chemical affinity: otherwise, translocation of molecules containing certain atoms like oxygen would fall out of the monotonic sequences.

### Temperature dependence

**Fig. 3:**
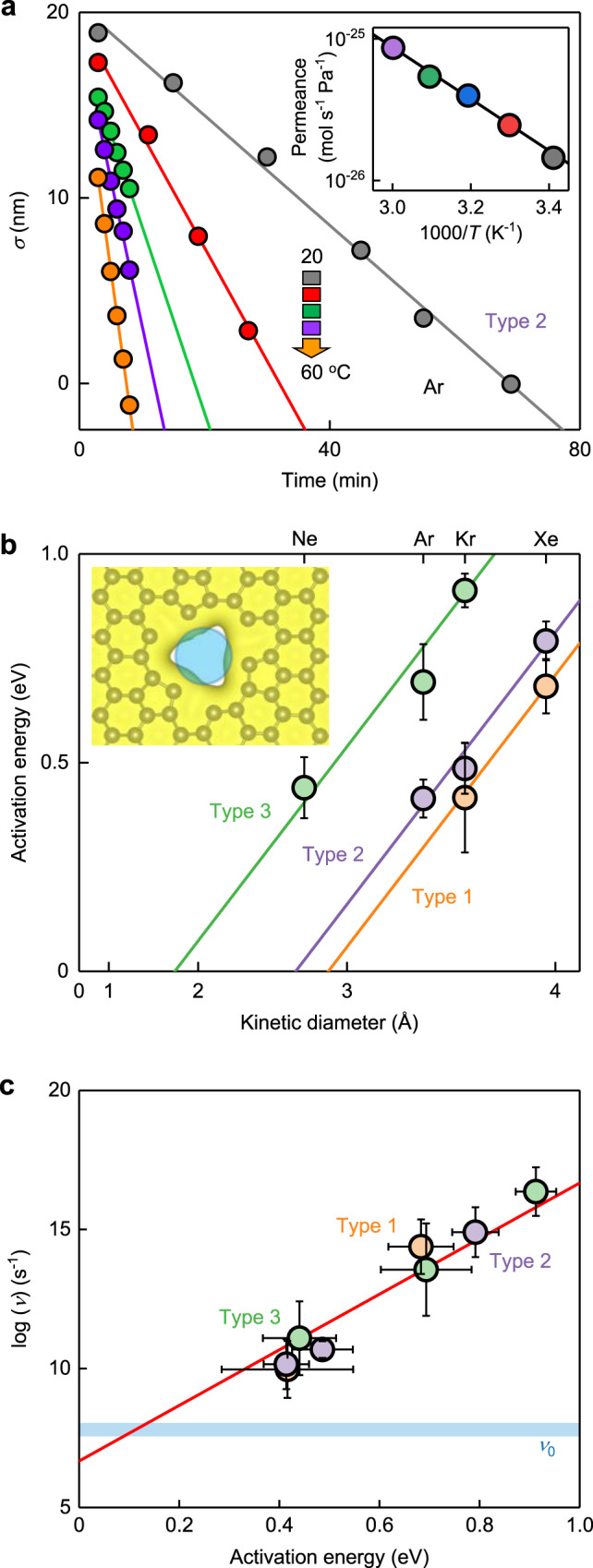
Characterizing the angstrom pores. **a** Example of the measured *T* dependences for type 2 pores (color coded *T*). Symbols: experimental data for Ar. Solid lines: linear fits. Inset: resulting Arrhenius plot (same color-coding). Solid curve: best fit yielding *E*_A_ ≈ 0.4 eV. **b** *E*_A_ for noble gases and different pore types shown as a function of *d*_K_ (note the nonlinear *x* axis). Symbols: experimental data with error bars showing SD, using the same set of devices as in Fig. 2. Solid curves: best fits with *E*_A_ = *α* (*d*_K_^2^ − *d*_0_^2^) using same *α*. Inset: One of possible atomic-scale defects (Supplementary Information) with *d*_0_ close to that of type 2 pores (blue circle’s diameter is 2.5 Å). **c** Impingement rates *ν* at 1 bar for the same gases and *E*_A_ as in **b**. The solid line: best fit using 1/*β* = 40 meV^34,35^. Blue shaded area: impingement rates *ν*_0_ if the noble atoms were coming from the bulk only. Note that, because of the upper limit on J* ≈ 10^−23^ mol s^−1^ Pa^−1^, we could not obtain the Arrhenius plots for gases with higher permeability than Ne. All *E*_A_ and impingement rates that were possible to obtain using our experimental setup are presented in **b** and **c**.

To investigate the underlying sieving mechanisms, we measured temperature (*T*) dependences of J* for all pore types. An example of such measurements is shown in Fig. 3a whereas Fig. 3b plots the extracted activation energies *E*_A_, using J* = *ν*/(*N*_A_*P*) exp(−*E*_A_/*k*_B_*T*) where *N*_A_ is the Avogadro number, *k*_B_ is the Boltzmann constant and *ν* is the impingement rate. If plotted as a function of *d*_K_^2^ (rather than *d*_K_) our data closely follow *E*_A_ = *α*(*d*_K_^2^ − *d*_0_^2^). This dependence allows the following interpretation. The pores have an empty space with the diameter *d*_0_ which is free from graphene’s electron clouds (inset of Fig. 3b). To “squeeze” through the pore, atoms and molecules must disturb a region of ~π(*d*_K_^2^ − *d*_0_^2^)/4 in size, and both electronic and elastic contributions are expected to scale with this area (Supplementary Fig. 5). The same *α* for all three pore types strongly supports the above interpretation, indicating that *α* is determined by the graphene properties, independently of pores’ configurations and diameters.

Next, we analyze the pre-exponential factors *ν* (Fig. 3c), which were found from the measured *T* dependences such as in Fig. 3a. For atoms arriving from the bulk, their impingement rate is given by *ν*_0_ = *AP*/(2π*mk*_B_*T*)^1/2^ where *A* is the effective pore area^5,17,18,21^, which yields *ν*_0_ of the order of 10^8^ s^−1^ at 1 bar for all our pores and gases. In contrast, the experiment yielded several orders of magnitude higher *ν* (Fig. 3c). This unambiguously indicates that translocating atoms come not from the bulk but mostly through adsorption and surface diffusion^17,18,20^.

## Discussion

The impingement rate *ν*_ad_ due to adsorption–diffusion processes can be expressed as (Supplementary Information)

1 $${\upsilon }_{{ad}}=\frac{P} {\sqrt{2{\pi} m{k}_{B}T}} \sqrt{\frac{{k}_{B}T}{2{\pi} m}}\frac{C}{{f}_{d}}$$

where *C* is the circumference of the pore and *f*_d_ is the desorption frequency of adsorbed gases. The desorption frequency *f*_d_ is described by the van ‘t Hoff equation: ${f}_{d}=\frac{{k}_{B}T}{h}{{{{{\rm{exp }}}}}}\left(\frac{\Delta S}{{k}_{B}}\right){{{{{\rm{exp }}}}}}\left(-\frac{{E}_{{ad}}}{{k}_{B}T}\right)$, where *h* is the Planck constant, *k*_B_*T*/*h* the vibration frequency of adsorbed gases, Δ*S* the entropy change during the permeation process and *E*_ad_ is the adsorption energy (*E*_ad_ is positive for this notation). The involvement of the adsorption–diffusion mechanism has the following consequences on gas selectivity^8,18^. First, the measured *E*_A_ should be notably lower than the actual translocation barriers, as the former values are reduced by the adsorption energy *E*_ad_ (Supplementary Information). Second, the mechanism should favor permeation of stronger-adsorbed diatomic gases, in agreement with their systematically higher J* as compared to noble gases (Fig. 2).

In the limit of zero *E*_A_, the impingement rate in Fig. 3c extrapolates close to *ν*_0_, as generally expected because this limit corresponds to the Knudsen flow. On the other hand, the strong dependence *ν* ≈ *ν*_0_ exp(*βE*_A_) in Fig. 3c is rather surprising. We speculate that it can be due to entropy loss during the surface-transport permeation process, as discussed in the literature^34,35^, and is a result of an increasingly large area that supplies gas molecules to the pore mouth, which rapidly grows with increasing the barrier^2,34^ (see Supplementary Information). Note that polymeric membranes exhibit similar *ν*(*E*_A_) dependences with a universal, material-independent coefficient *β* ≈ 1/(40 meV)^34,35^ which value also matches well our results (Fig. 3c). The origins of such universality remain unknown^34,35^. Although the importance of the adsorption–diffusion mechanism for small pores is well documented in the literature^17,19,34,36^, it is especially difficult to extrapolate the existing simulations onto our case because of the extreme crowding effects expected for ultimately small, angstrom-scale pores^19^. The surface contamination of any realistic membrane (rather than idealized graphene) complicates perspective theoretical analysis even further.

To conclude, our work provides experimental feedback for extensive theoretical studies of molecular transport through angstrom-scale pores and reveals some unexpected features of the activated-transport mechanism. The mechanism critically involves adsorption and surface diffusion, which places strong constraints on the pore sizes required to reach high selectivity. The found pre-exponential factor ∝exp(*βE*_A_) counteracts the Arrhenius behavior exp(−*E*_A_/*k*_B_*T*) and strongly reduces selectivity for any given pair of gases. Although atomic structures of the studied pores remain unknown, type 3 pores could be similar in size to hepta-vacancies (Supplementary Information) and intrinsic pores in *γ*-graphyne. Only if 2D membranes with such angstrom pores of high density are developed, one can envisage separation technologies with selectivities beyond the existing selectivity-permeability bounds (for projections based on our results, see Supplementary Fig. 6).

## Methods

### Device fabrication and inspection

To make our devices and test their atomically tight sealing, we followed the procedures developed in ref. ^21^. In brief, monocrystals of graphite with a thickness of >200 nm were prepared by mechanical exfoliation on an oxidized silicon wafer. The crystals were examined in an optical microscope using both dark-field and differential-interference-contrast modes to locate relatively large areas (over tens of microns in size), which were free from wrinkles, folds, atomic-step terraces, and other defects. Then, using electron-beam lithography and dry etching, an array of microwells with internal diameters of 1–3 μm and depth of ∼100 nm was fabricated within the found atomically flat areas. After overnight annealing at 400 °C in H_2_/Ar atmosphere (volume ratio of 1:10), the microwells were sealed with a large crystal of monolayer graphene, which was transferred in ambient air (Fig. 1).

The resulting devices were carefully inspected using AFM, and those showing any damage to their sealing were discarded. Such damage could be, for example, extended defects in the atomically flat top surface of the microwells or wrinkles in the graphene sealing^21^. The remaining devices were leak tested by placing them into a stainless-steel chamber containing Ar or Kr at a partial pressure *P* ≈ 3 bar. After a few days, they were taken out and quickly (typically within 3 min) checked using AFM for any changes in the membrane position (Fig. 1b). Again, we discarded those devices that exhibited any sign of leakage, namely, if changes in the membrane position after pressurization were >1 nm. Finally, we repeated the same leak test but in an atmosphere of helium at 1 bar. Only devices with no changes in membrane positions were kept for further investigation.

### Perforating graphene with low-energy electrons

Devices that successfully passed the above inspection were exposed to electron irradiation in scanning electron microscope Zeiss EVO. To evaluate the radiation exposure of the studied graphene membranes, we first measured the beam current using a Faraday cup. Then the electron beam was switched off and the membrane device with the known coordinates on the substrate was moved into a central position within a projected exposure area. The beam was then switched on and scanned over this entire area for a few seconds, using magnification 700 with a single area scan lasting ∼0.1 s. The simultaneously taken images ensured that membranes were in the center and properly exposed to the beam. After each exposure, the devices were subjected to the same leak tests as described above. We repeated the exposure-test cycle several times until the irradiated container started to exhibit a leak, indicating a defect created in the graphene membrane. In about 20% of cases, we could not create any discernible leak, no matter how long the graphene membranes were exposed to the electron beam. In another 20% of cases, we found an increase in permeation after additional exposures, which probably indicates the creation of the second, larger defect (Supplementary Fig. 3). No changes in permeation rates occurred after further prolonged exposures, even those leading to visible hydrocarbon contamination^25–27,37^.

## Supplementary information

Supplementary Information

## Data Availability

All relevant data to support this study are available upon request from the corresponding authors.
