# Supplementary material for: Exponentially selective molecular sieving through angstrom pores
Source: Nat Commun. 2021 Dec 9;12:7170. doi: 10.1038/s41467-021-27347-9 (PMC8660907; doi:10.1038/s41467-021-27347-9)
Supplement: Supplementary file 1 — Supplementary Information [file 41467_2021_27347_MOESM1_ESM.pdf]

# Supplementary Information

P. Z. Sun, M. Yagmurcukardes, R. Zhang, W. J. Kuang, M. Lozada-Hidalgo, B. L. Liu, H.-M. Cheng, F. C. Wang, F. M. Peeters, I. V. Grigorieva, A. K. Geim

**This file contains**

**Supplementary Notes (1-6)**

1. Evaluation of permeation rates.
2. Sealing the pores with Au nanoparticles.
3. Effect of high radiation doses.
4. Permeation dominated by surface-adsorbed gases.
5. Ab-initio simulations for gas translocation through graphene pores.
6. Comparison with other gas-selective membranes.

**Supplementary Figures (1-6)**

Supplementary Figure 1. Pressure dependence.
Supplementary Figure 2. Sealing the angstrom pores in graphene.
Supplementary Figure 3. Effect of additional irradiation.
Supplementary Figure 4. Impingement rate rapidly increases for stronger adsorbing atoms.
Supplementary Figure 5. Simulations of gas permeation through graphene pores.
Supplementary Figure 6. Projected performance of angstroporous 2D materials.

**Supplementary References (1-105)**

**Supplementary Notes**

1. Evaluation of permeation rates. The permeation rates $J$ were evaluated using the initial linear slopes of $\sigma(t)$ (see Fig. 1d) following the approach detailed in refs. 1-3. Briefly, the pressure inside the microcavities is the sum of the atmospheric pressure $P_a$ coming from the trapped air and the initial partial pressure $P(0)$ of the tested gas. The resulting differential pressure $P$ acting on a graphene membrane is given by Hencky's solution for a clamped circular membrane[4]

$$P(t) = \frac{\kappa Y L \sigma(t)^3}{a^4} \tag{S1}$$

where $\kappa \approx 3$ is a coefficient that depends on Poisson's ratio[2], $Y$ is Young's modulus, $L$ is the membrane thickness, and $a$ is the radius of the cavity with a depth $H$. According to the ideal gas law

$$(P_a + P)V = (n_{air} + n_{gas})N_A k_B T \tag{S2}$$

where $n_{air}$ and $n_{gas}$ are the number of moles of air and tested gas inside the container, respectively, and $N_A$ is the Avogadro constant. The total volume of the trapped gas $V = \pi a^2 H + c\pi a^2 \sigma$ is given by microwell's volume, $\pi a^2 H$, and the additional volume $c\pi a^2 \sigma$ due to the bulging membrane, where $c \approx 0.5$ is a geometric constant accounting for the membrane curvature. For simplicity, we neglect the volume's reduction caused by sagging and, for the purpose of this section only, count $\sigma$ from its unpressurized state ($P = 0$) rather than the top graphite surface.

By differentiating eq. S2 while utilizing eq. S1, we obtain

$$J \equiv -\frac{dn_{gas}}{dt} \approx -\frac{\pi a^2}{N_A k_B T}\frac{d}{dt}(P_a c\sigma + PH + Pc\sigma) = \frac{\pi a^2}{N_A k_B T}(P_a c + 3PH/\sigma + 4Pc)\left|\frac{d\sigma}{dt}\right| \tag{S3}$$

where we also used the fact that, because of the equal partial pressures of air outside and inside the container, there is little flow of air through the pore, $\frac{dn_{air}}{dt} \approx 0$. The flow rate $J$ on the left-hand side of eq. S3 is generally proportional to $P$ and, therefore, $\propto \sigma^3(t)$ as per eq. S1, whereas the second and third terms on the right-hand side are proportional to $\sigma^2$ and $\sigma^3$, respectively. Accordingly, eq. S3 is a nonlinear but autonomous differential equation. It allows an analytical solution $\sigma(t)$ but, because we are interested only in the initial response, it is straightforward to show that, initially, $\sigma$ evolves linearly with time, $\frac{d\sigma}{dt}(0)=$ *const.* The solution is given by eq. S3 at $t = 0$, that is,

$$J(0) = \frac{\pi a^2}{N_A k_B T}\left[P_a c + \frac{3P(0)H}{\sigma(0)} + 4P(0)c\right]\left|\frac{d\sigma}{dt}(0)\right| \tag{S4}$$

Generally, the linearity should hold only for small changes in $\sigma$, but our modelling using the full solution of eq. S3 shows that, if $P(0)$ is above a few bars, the fitting of the experimental curves with a linear dependence using eq. S4 should result in $J$ accurate within a factor of < 2, even if $\sigma$ changes by as much as 40%. Furthermore, for our usual case of $P(0) > P_a$ and $H > \sigma$, the first term in the brackets in eq. S4 can be neglected and, therefore, the permeation rates should depend linearly on $P$, which is consistent with our assumption above and agrees with the experiment (Supplementary Fig. 1). This also allows us to introduce

the permeance $J^* = J/P$, which characterizes the pores, independently of $P(0)$ used for pressurization.

_2. Sealing the pores with Au nanoparticles._ The earlier work[3] had used 'leaky' containers made from oxidized Si wafers to evaluate permeation rates through individual pores obtained by exposure of graphene membranes to oxygen under UV radiation. One of the experimental proofs invoked in ref. 3 to argue the presence of extremely rare, possibly individual pores – rather than their distribution – was the sealing of those pores with Au nanoparticles. For completeness of our studies, we repeated the same experiments and observed the same behavior as reported in ref. 3. To this end, we deposited a small amount of Au on top of our containers exhibiting the characteristic leak rates after their electron-beam irradiation. A single cycle of Au deposition lasted for 3 s at a rate of 0.3 Å $s^{-1}$, which resulted in nanoparticles covering < 1% of the membrane area, in good agreement with the literature and observations in ref. 3. Then the devices were pressurized with Ar, and $\sigma(t)$ was monitored by AFM. This procedure was repeated several times. As exemplified in Supplementary Fig. 2, we typically observed a sharp decrease in $J^*$ after one or two deposition cycles (> 95% of cases), and $J^*$ remained essentially constant after further cycles. Although this experiment alone does not present unequivocal proof of individual pores present in our membranes, it adds to the other, much stronger evidence described in the main text.

_3. Effect of high radiation doses._ Several studied devices with pores identified as type 3 were exposed to further electron-beam radiation at 8 keV. Most of them (> 80%) exhibited stable $J^*$ even after radiation doses of 100 times larger (Supplementary Fig. 3). This indicated that no additional pores appeared and no modification to the existing type-3 pores occurred. Only on a few occasions, we observed a notable increase in permeation rates, which fell into the range of $J^*$ typical for type-2 pores (green curves in Supplementary Fig. 3). We speculate that such pores developed from the original type-3 pores or, alternatively, one extra pore appeared at another weak spots in the membranes. Statistically, it is quite plausible that, if our devices developed mostly a single pore whereas ~20% of them developed none, there would be a few membranes with two weak spots.

_4. Permeation dominated by surface-adsorbed gases._ Permeating gas particles could either come from the bulk phase, striking directly at pore's area $A$, or diffuse into pore's mouth after first being adsorbed on graphene within a finite-size area $A_{fin}$ around the pore. Accordingly, the permeation rate can be expressed as $J = J_{bu} + J_{ad}$ where $J_{bu}$ and $J_{ad}$ denote the bulk and adsorbed-phase contributions, respectively. The former is described by[5]

$$J_{bu} = \frac{\nu_0}{N_A} \exp\left(-\frac{E}{k_B T}\right) = \frac{A}{N_A} \frac{P}{\sqrt{2\pi m k_B T}} \exp\left(-\frac{E}{k_B T}\right) \quad \text{(S5)}$$

where $\nu_0$ is the impingement rate from the bulk phase, that is, the number of gas atoms or molecules striking the pore area each second. Similarly, the permeation rate from the adsorption phase can be written as

$$J_{ad} = \frac{\nu_{ad}}{N_A} \exp\left(-\frac{E}{k_B T}\right) \quad \text{(S6)}$$

where $\nu_{ad}$ is the impingement rate for adsorbed atoms or molecules, which can in turn be expressed as[6]

$$v_{ad} = \sqrt{\frac{k_B T}{2\pi m}}\rho C \tag{S7}$$

where $\rho$ is the areal density of adsorbed gases and $C$ is the pore circumference, $C \approx \pi d_P$. In this equation, it is assumed that absorbed atoms or molecules form an ideal 2D gas on the graphene surface owing to their little interaction with atomic corrugations[7]. The areal density can be expressed[5] as $\rho \approx \frac{P}{\sqrt{2\pi m k_B T}}\frac{1}{f_d}$ where the desorption frequency $f_d$ can be written as[8,9] $f_d = f_0 K$ with $f_0 = k_B T/h$ being the frequency of molecular vibration and $h$ the Planck constant. The thermodynamic equilibrium constant $K$ is given by the van 't Hoff equation[8,9]

$$K = \exp\left(\frac{\Delta S}{k_B}\right)\exp\left(-\frac{E_{ad}}{k_B T}\right) \tag{S8}$$

where $\Delta S$ is the entropy change during the permeation process and $E_{ad}$ is the adsorption energy (in this form, $E_{ad}$ is positive). Combining the above equations, we obtain

$$J = \frac{1}{N_A}(v_0 + v_{ad})\exp\left(-\frac{E}{k_B T}\right) = \frac{1}{N_A}\frac{P}{\sqrt{2\pi m k_B T}}\left(A + \sqrt{\frac{k_B T}{2\pi m}}\frac{C}{f_d}\right)\exp\left(-\frac{E}{k_B T}\right) \tag{S9}$$

which is consistent with the linear $P$ dependence observed experimentally. The measured $T$ dependences allowed us to evaluate both impingement rates $\nu$ and the activation energy $E_A$ (see the main text). The found $\nu$ (Fig. 3c) were many orders of magnitude larger than $\nu_0$, indicating that the bulk phase contributes little to the observed permeation. Accordingly, eq. S9 can be simplified and further extended as

$$J = \frac{v_{ad}}{N_A}\exp\left(-\frac{E}{k_B T}\right) = \frac{P d_P h}{2 N_A m k_B T}\exp\left(-\frac{\Delta S}{k_B}\right)\exp\left(-\frac{E - E_{ad}}{k_B T}\right) \tag{S10}$$

According to eq. S10, the permeation barrier $E$ should be larger than the measured activation energy $E_A = E - E_{ad}$. Furthermore, adsorbed atoms or molecules diffusing along the graphene surface and permeating through angstrom-scale pores are expected[10-12] to exhibit a considerable entropy loss $\Delta S < 0$ at the translocation position, which results in an enhancement of $J$ by a factor of $\exp(|\Delta S|/k_B)$, similar to the case of polymeric membranes[13,14]. Supplementary Fig. 4 shows the measured impingement rate $\nu$ as a function of the adsorption energy $E_{ad}$[15]. One can see a clear correlation between the two, for all pore types. Basically, the heavier the atom, the higher its adsorption energy and the impingement rates. This observation agrees with eq. S10. The impingement rate is expected to depend exponentially on entropy changes, $\Delta S$. The latter increase with increasing the adsorption energy (crowding effect).

*5. Ab-initio simulations for gas translocation through graphene pores.* The adsorption energy and diffusion barriers for noble gases on graphene are much smaller than the found activation energies[7,15], which suggests that translocation is the rate-limiting step. Trying to evaluate the energy barrier $E$ for translocation of a noble atom through graphene pores, we performed first-principles calculations in the framework of density functional theory (DFT), as implemented in the Vienna *ab-initio* simulation package (VASP)[16]. The Perdew-Burke-Ernzerhof (PBE) form of generalized gradient approximation (GGA) was adopted to describe the electron exchange and correlation[17]. The van der Waals (vdW) correction to the

GGA functional was included using the DFT-D2 method of Grimme.

To construct graphene pores of different sizes, a certain number of carbon atoms were removed from the graphene lattice followed by bond reconstruction to eliminate undercoordination of the edge atoms. To be specific, Stone Wales (SW or 57) defect, 585- and 555-777- divacancies, tetra- and hexa- vacancies were created based on a 5×5 graphene supercell, whereas the largest deca-vacancy was created in a 6×6 supercell. The pore area obviously increased with the number of removed carbon atoms. For example, the largest defect sites (pores) for SW and 555-777-divacancy were 7-membered rings, whereas those in 585-divacancy, tetra-, hexa- and deca- vacancies were 8-, 9-, 10- and 12-membered rings, respectively. As an example, the deca-vacancy is shown in Supplementary Fig. 5a. The geometric areas $A_n$ of these pores as determined by the position of carbon atoms on the edges (where $n$ is the number of edge atoms) were $A_7 \approx 7.6$ and 7.9 Å$^2$ for SW and 555-777-divacancy, $A_8 \approx 11.0$ Å$^2$ for 585-divacancy, $A_9 \approx 14.3$ Å$^2$ for tetra-vacancy, $A_{10} \approx 17.1$ Å$^2$ for hexa-vacancy and $A_{12} \approx 28.0$ Å$^2$ for deca-vacancy. However, because of the electronic clouds surrounding carbon atoms, the penetrating gas atoms should effectively experience considerably smaller openings in the graphene lattice.

It is instructive to separate electronic and elastic contributions into the energy barrier $E$. To this end, we first fixed the positions of carbon atoms around the pores (fixed configuration). In this case, the barrier is fully determined by distortions of the electron density in graphene, which is required for gas atoms to squeeze through. Next, we allowed the lattice to be flexible in all three directions (flexible configuration), which yielded the combined effect of the electron-density and elastic lattice distortions. For details of our numerical simulations, we refer to the previous report[18]. We found that, in both fixed and flexible configurations, the barrier $E$ for gas transport through all the studied pores scaled well with $d_K^2$, in agreement with our experiment. All non-reconstructed pores with small $A_n$ ($n$ = 7-12) yielded $E$ considerably higher than $E_A$ found experimentally. For example, in its non-reconstructed state (Supplementary Fig. 5a), the deca-vacancy provided $E \approx 1.5$, 2.2 and 3.5 eV for Ar, Kr and Xe atoms, respectively. As the next step, the vacancy configurations were allowed to fully relax in all three directions. We found that small pores remained approximately circular. In contrast, our largest pore (deca-vacancy) experienced notable edge reconstruction, as illustrated in Supplementary Fig. 5b. This resulted in its larger pore size ($A \approx 46.8$ Å$^2$) and reduced stress. We found that $E$ through this reconstructed pore were close to experimental $E_A$ for type-1 and type-2 pores, regardless of whether the positions of rim atoms were fixed or flexible during translocation (Supplementary Fig. 5d). In comparison with its fixed configuration, the pore shown in Supplementary Fig. 5b exhibited a reduction in $E$ of < 11% for the flexible configuration and the largest simulated atom, Xe. This gives a general idea about the amount of elastic energy involved in translocation of gases through atomic-scale pores. Using the same approach, we also constructed an intermediate size pore by removing 7 carbon atoms and, after full relaxation, the resulting hepta-vacancy (Supplementary Fig. 5c) exhibited the geometric area $A \approx 38.7$ Å$^2$. In its flexible configuration, the calculated barriers $E$ closely matched $E_A$ found for type-3 pores in our experiments (Supplementary Fig.

5d).

The number of possible pore configurations increases rapidly, exponentially with the number of atoms removed[19]. For example, about 100 different configurations are expected if 10 atoms were removed, leaving aside effects of functionalization of pore edges, which is most probable for realistic pores. This makes it essentially impossible to determine the atomic structure of even our smallest, type-3 pores by comparing their permeations rates with theory and simulations. Accordingly, the three different vacancies (7 and 10 atoms removed) in Supplementary Fig. 5 should be considered only as a toy model to infer the approximate pore size that is needed for the reported exponential selectivity. Our model is also informative in allowing us to understand the found quadratic dependence of the energy barrier on kinetic diameter.

*6. Comparison with other gas-selective membranes.* Our results allow comparison of potential performance of angstroporous 2D materials with that of membranes made from 3D materials. To this end, let us first assume a pore density of $10^{14}$ $cm^{-2}$ or one pore per $nm^2$. Such a high density is probably unachievable by top-down approaches of introducing defects in graphene (for example, sub-nm pores with densities of ~$10^{12}$ $cm^{-2}$ were demonstrated in graphene using ion bombardment[20]). Much higher densities are offered by several angstroporous graphene allotropes[21-23] which can potentially be obtained by growth (bottom-up approach). These two projections for top-down and bottom-up approaches are considered separately below. To allow comparison between 2D and 3D cases, we also assume for simplicity that all 3D membranes can be made down to ~100 nm in thickness, even though this is rather optimistic and impossible for many of the materials without loss of functionality[24-26]. The results of our analysis are summarized in Supplementary Fig. 6 that plots the experimentally observed selectivities as a function of permeance for 11 different pairs of gases (namely, $O_2/N_2$, $H_2/N_2$, $He/N_2$, $He/H_2$, $CO_2/N_2$, $H_2/CO_2$, $He/CO_2$, $CO_2/CH_4$, $H_2/CH_4$, $He/CH_4$ and $N_2/CH_4$). The literature data include membranes made from polymers[27-44], metal-organic frameworks[45-55], graphene oxide laminates[56-65], covalent organic frameworks[66-69], zeolites[70-78], transition metal dichalcogenides[79-83], MXenes[84,85], layered double hydroxides[86,87], carbon nitride[88], silica[89-92], silicon carbide[93] and carbon molecular sieves[94-97]. The figure also shows the results previously reported for porous graphene[98-104] and the current Robeson bounds for polymeric membranes[105].

One can see in Supplementary Fig. 6 that angstroporous 2D materials obtained by growth techniques can in principle provide performance that compares favorably with all the other membranes studied for gas separation. Particularly, angstroporous membranes can provide orders-of-magnitude improvements for separation and removal of relatively large gas molecules (such as $CH_4$ in the case of Supplementary Fig. 6 and Xe). This ability is particularly prominent for our smallest, type-3 pores that essentially block these molecules. Even at densities of ~$10^{10}$–$10^{12}$ $cm^{-2}$, which could be achievable by top-down approaches, graphene with type-3 or similar-size pores can offer superior performance, owing to the exponentially-high selectivity for gas molecules with $d_K$ differing by only a factor of ~2. The sieving effect is less pronounced for smaller molecules, which shows that angstroporous 2D membranes offer notable advantages mostly in the activation regime ($d_P < d_K$). To achieve better selectivity-permeability tradeoff

with respect to smaller molecules ($d_K$ < 3.5 Å such as $O_2$ and $CO_2$ in Supplementary Fig. 6), pores smaller than type-3 are required ($d_P \leq 1.5$ Å). Those are potentially available in some graphynes[21-23] but have not been demonstrated so far in experiment.

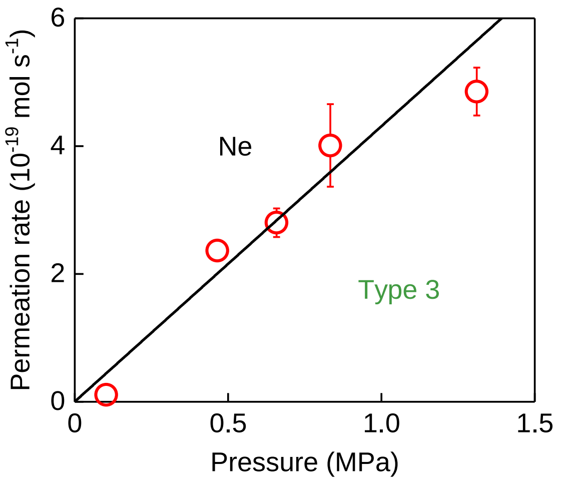


**Supplementary Figure 1| Pressure dependence.** Permeation rates for Ne at different *P* using a type-3 pore. Symbols: experimental data. Solid curve: best linear fit. Error bars: SD for linear-in-time fitting of $\sigma$ and are shown only if larger than symbols.

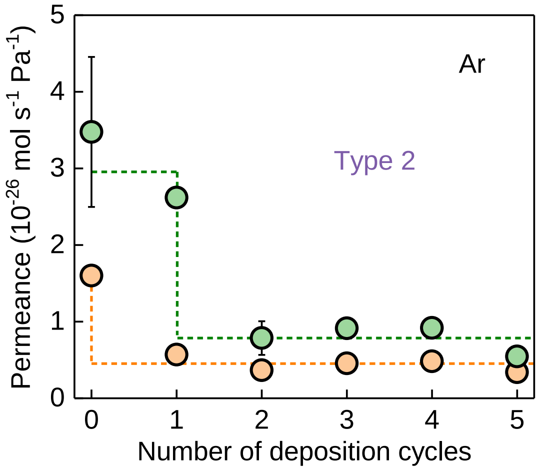


**Supplementary Figure 2| Sealing the angstrom pores in graphene.** Permeance of Ar through type-2 pores during repeated deposition of Au nanoparticles (colors mark two different devices). The error bars are SD for linear fits of $\sigma(t)$. Dashed curves: guides to the eye. Note that Au nanoparticles placed on top of pores do not seal them completely, and $J^*$ is typically suppressed by a factor of ~4, close to the observations reported in ref. 3.

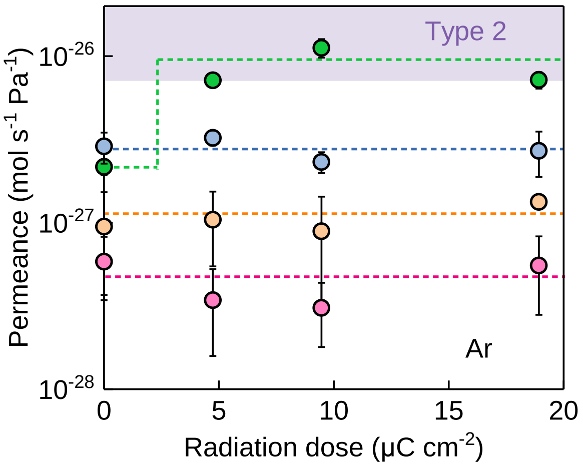


**Supplementary Figure 3| Effect of additional irradiation.** Initially all the devices (different colors) had the smallest (type-3) pores created by doses of <0.5 μC cm$^{-2}$. Then the graphene membranes were subjected to further electron radiation (doses were up to 100 times higher). Less than 20% of pores exhibited discernable changes in their permeation rates, as exemplified by the device shown in green. Its pore seemed to evolve from type 3 to type 2. Symbols: experimental data with error bars indicating SD for the linear $\sigma(t)$ fits. Dashed lines: guides to the eye. The shaded area refers to the range of Ar permeances observed for type-2 pores.

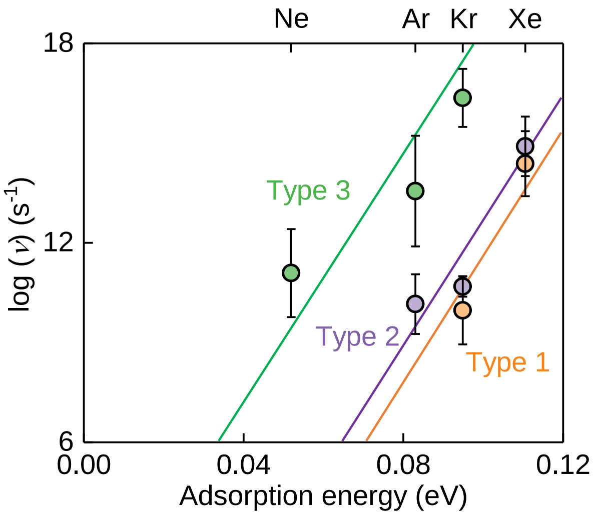


**Supplementary Figure 4| Impingement rate rapidly increases for stronger adsorbing atoms.** Symbols: $\nu$ from Fig. 3c of main text as a function of the adsorption energy (data from ref. 15). Solid lines: guides to the eyes. Same color coding as in Figs. 3b, c.

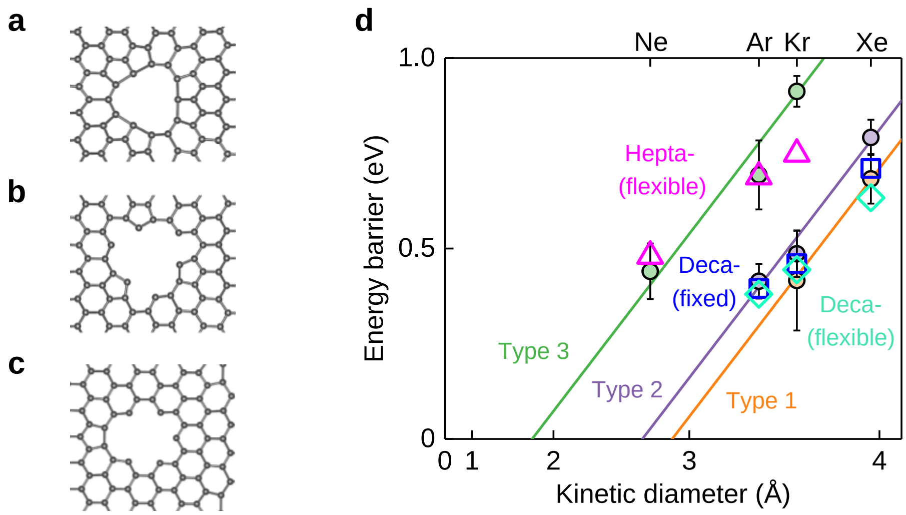


**Supplementary Figure 5| Simulations of gas permeation through graphene pores.** **a**, **b**, Schematics for the pore formed by removing 10 carbon atoms in its initial (**a**) and reconstructed (**b**) configurations. **c**, Pore formed by removing 7 carbon atoms (shown is its reconstructed configuration). **d**, The barriers $E$ for translocation of Ne, Ar, Kr and Xe through the reconstructed pores in (**b**, **c**). During translocation, carbon atoms of the deca-vacancy were either fixed (blue squares) or allowed to elastically deform (cyan diamonds). Pink triangles: $E$ for deformable hepta-vacancy. The calculated $E$ are compared with $E_A$ found experimentally (color-coded symbols and lines from Fig. 3b of the main text).

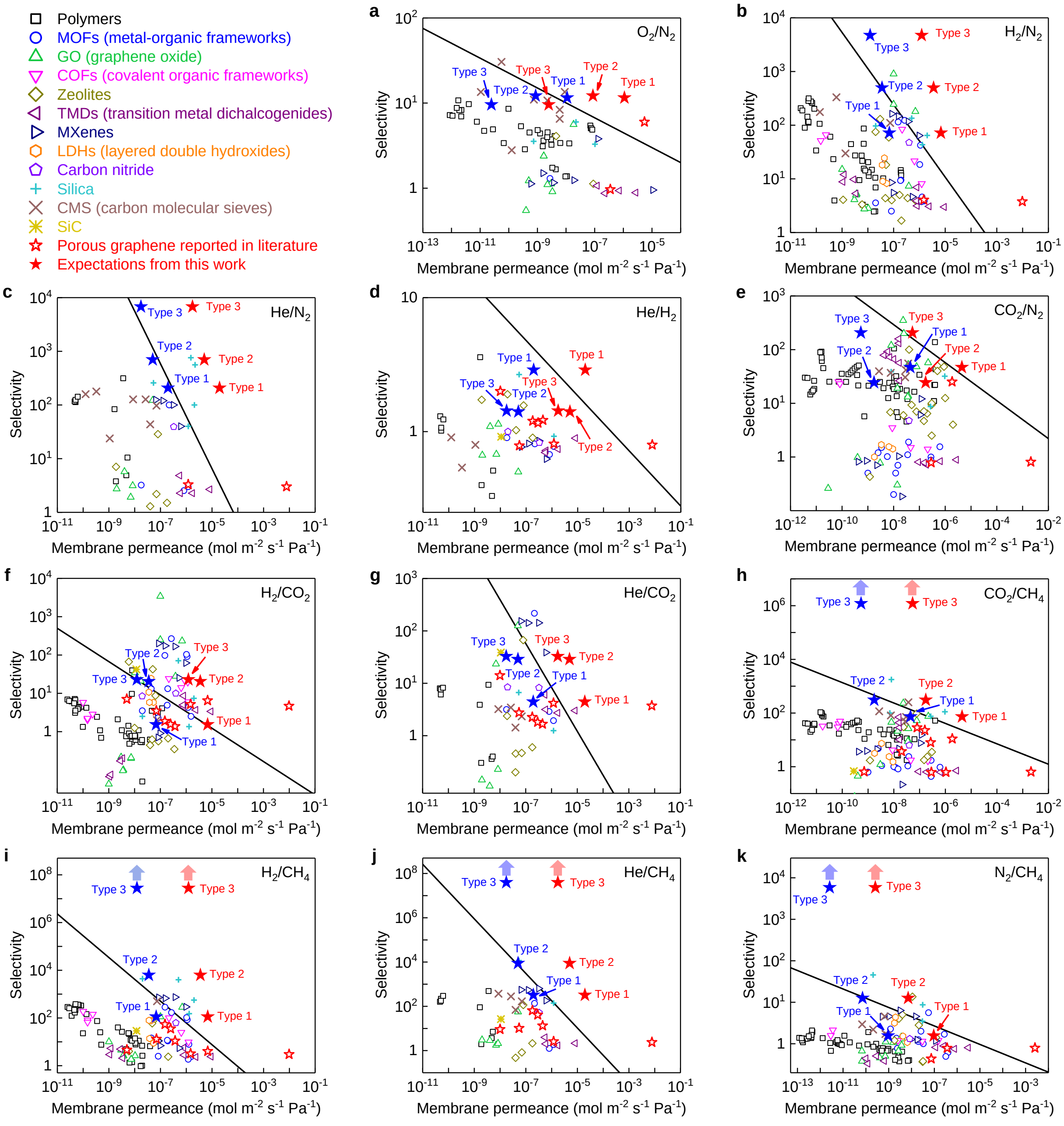


**Supplementary Figure 6| Projected performance of angstroporous 2D materials.** (**a**-**k**) Selectivity for different pairs of gases as stated in the upper right corners of each panel. The solid-star symbols are for 2D membranes that are assumed to have type 1, 2 or 3 pores with densities of $10^{12}$ (blue) and $10^{14}$ $cm^{-2}$ (red), which could be obtained by top-down and bottom-up techniques, respectively. Empty stars: projected performances reported previously for porous graphene membranes. The other symbols are literature data as specified in the figure (top left). The black lines are the current Robeson bounds for polymers assuming their 100 nm thickness (adapted from ref. 105). The arrows in (**h**-**k**) indicate that we

can provide only the minimal bounds for type-3 pores because of the limits on permeation of $CH_4$ and Xe through these pores.

## Supplementary references


1. Sun, P. Z. et al. Limits on gas impermeability of graphene. *Nature* **579**, 229–232 (2020).
2. Koenig, S. P., Wang, L., Pellegrino, J. & Bunch, J. S. Selective molecular sieving through porous graphene. *Nat. Nanotechnol.* **7**, 728–732 (2012).
3. Wang, L. et al. Molecular valves for controlling gas phase transport made from discrete ångström-sized pores in graphene. *Nat. Nanotechnol.* **10**, 785–790 (2015).
4. Hencky, H. Uber den spannungzustand in kreisrunden platten mit verschwindender biegungssteiflgeit. *Z. Math. Phys.* **63**, 311–317 (1915).
5. Landau, L. D. & Lifshitz, E. M. Course of theoretical physics Vol. 5 Statistical physics 3rd edn (Pergamon Press, 1980).
6. Yuan, Z., Misra, R. P., Rajan, A. G., Strano, M. S. & Blankschtein, D. Analytical prediction of gas permeation through graphene nanopores of varying sizes: understanding transitions across multiple transport regimes. *ACS Nano* **13**, 11809–11824 (2019).
7. Bartolomei, M., Carmona-Novillo, E., Hernández, M. I., Campos-Martínez, J. & Pirani, F. Global potentials for the interaction between rare gases and graphene-based surfaces: an atom–bond pairwise additive representation. *J. Phys. Chem. C* **117**, 10512–10522 (2013).
8. Eyring, H. The activated complex in chemical reactions. *J. Chem. Phys.* **3**, 107–115 (1935).
9. Hanggi, P., Talkner, P. & Borkovec, M. Reaction-rate theory: fifty years after Kramers. *Rev. Mod. Phys.* **62**, 251–342 (1990).
10. Campbell, C. T. & Sellers, J. R. The entropies of adsorbed molecules. *J. Am. Chem. Soc.* **134**, 18109–18115 (2012).
11. Dauenhauer, P. J. & Abdelrahman, O. A. A universal descriptor for the entropy of adsorbed molecules in confined spaces. *ACS Cent. Sci.* **4**, 1235–1243 (2018).
12. Meares, P. The diffusion of gases through polyvinyl acetate. *J. Am. Chem. Soc.* **76**, 13, 3415–3422 (1954).
13. Freeman, B. D. Basis of permeability/selectivity tradeoff relations in polymeric gas separation membranes. *Macromolecules* **32**, 375–380 (1999).
14. Robeson, L. M., Freeman, B. D., Paul, D. R. & Rowe, B. W. An empirical correlation of gas permeability and permselectivity in polymers and its theoretical basis. *J. Membr. Sci*. **341**, 178–185 (2009).
15. Shepard, R., Shepard, S. & Smeu, M. Ab initio investigation into the physisorption of noble gases on graphene. *Surf. Sci.* **682**, 38–42 (2019).
16. Gresse, K. & Furthmuller, F. Efficient iterative schemes for ab initio total-energy calculations using a plane-wave basis set. *Phys. Rev. B* **54**, 11169 (1996).
17. Perdew, J. P., Burke, K. & Ernzerhof, M. Generalized gradient approximation made simple. *Phys. Rev. Lett.* **77**, 3865–3868 (1996).

18. Griffin, E. et al. Proton and Li-ion permeation through graphene with eight-atom-ring defects. *ACS Nano* **14**, 7280–7286 (2020).
19. Govind Rajan, A., et al. Addressing the isomer cataloguing problem for nanopores in two-dimensional materials. *Nat. Mater.* **18**, 129–135 (2019).
20. O'Hern, S. C. et al. Selective ionic transport through tunable subnanometer pores in single-layer graphene membranes. *Nano Lett.* **14**, 1234−1241 (2014).
21. Qiu, H., Xue, M., Zhang, Z. & Guo, W. Graphynes for water desalination and gas separation. *Adv. Mater.* **31**, 1803772 (2019).
22. Gao, X., Liu, H., Wang, D. & Zhang, J. Graphdiyne: synthesis, properties, and applications. *Chem. Soc. Rev.* **48**, 908–936 (2019).
23. Neumann, C. et al. Bottom-up synthesis of graphene monolayers with tunable crystallinity and porosity. *ACS Nano* **13**, 7310−7322 (2019).
24. Wang, L. et al. Fundamental transport mechanisms, fabrication and potential applications of nanoporous atomically thin membranes. *Nat. Nanotechnol.* **12**, 509–522 (2017).
25. Epsztein, R., DuChanois, R. M., Ritt, C. L., Noy, A. & Elimelech, M. Towards single-species selectivity of membranes with subnanometre pores. *Nat. Nanotechnol.* **15**, 426–436 (2020).
26. Park, H. B., Kamcev, J., Robeson, L. M., Elimelech, M. & Freeman, B. D. Maximizing the right stuff: the trade-off between membrane permeability and selectivity. *Science* **356**, eaab0530 (2017).
27. Li, S., Wang, Z., Yu, X., Wang, J. & Wang, S. High-performance membranes with multi-permselectivity for $CO_2$ separation. *Adv. Mater.* **24**, 3196–3200 (2012).
28. Shen, Y., Wang, H., Liu, J. & Zhang, Y. Enhanced performance of a novel polyvinyl amine/chitosan/graphene oxide mixed matrix membrane for $CO_2$ capture. *ACS Sustain. Chem. Eng.* **3**, 1819–1829 (2015).
29. Fu, Q. et al. A novel cross-linked nano-coating for carbon dioxide capture. *Energy Environ. Sci.* **9**, 434–440 (2016).
30. Du, N. et al. Polymer nanosieve membranes for $CO_2$-capture applications. *Nat. Mater.* **10**, 372–375 (2011).
31. Park, H. B. et al. Polymers with cavities tuned for fast selective transport of small molecules and ions. *Science* **318**, 254–258 (2007).
32. Shan, M. et al. Facile manufacture of porous organic framework membranes for precombustion $CO_2$ capture. *Sci. Adv.* **4**, 1698–1705 (2018).
33. Carta, M. et al. An efficient polymer molecular sieve for membrane gas separations. *Science* **339**, 303–307 (2013).
34. Rezac, M. E. & Schöberl, B. Transport and thermal properties of poly(etherimide)/acetylene terminated monomer blends. *J. Memb. Sci.* **156**, 211–222 (1999)
35. Nagai, K., Higuchi, A. & Nakagawa, T. Gas permeability and stability of poly(1-trimethylsilyl-1-propyne-co-1-phenyl-1-propyne) membranes. *J. Polym. Sci., B, Polym. Phys.* **33**, 289–298 (1995).
36. Hamid, M. A., Chung, Y. T., Rohani, R. & Junaidi, M. U. Miscible-blend polysulfone/polyimide membrane for hydrogen purification from palm oil mill effluent fermentation. *Sep. Purif. Technol.* **209**, 598–607 (2019).

37. Han, J. Y., Lee, W. S., Choi, J. M., Patel, R. & Min, B. R. Characterization of polyethersulfone/polyimide blend membranes prepared by a dry/wet phase inversion: Precipitation kinetics, morphology and gas separation. *J. Membr. Sci.* **351**, 141–148 (2010).
38. Asghar, H., Ilyas, A., Tahir, Z., Li, X. & Khan, A. L. Fluorinated and sulfonated poly (ether ether ketone) and Matrimid blend membranes for $CO_2$ separation. *Sep. Purif. Technol.*, 203, 233–241 (2018).
39. Giel, V. et al. Polyaniline/polybenzimidazole blends: Characterisation of its physico-chemical properties and gas separation behaviour. *Eur. Polym. J.* **77**, 98–113 (2016).
40. Yong, W. F., Li, F. Y., Chung, T. S. & Tong, Y. W. Molecular interaction, gas transport properties and plasticization behavior of cPIM-1/Torlon blend membranes. *J. Membr. Sci.* **462**, 119–130 (2014).
41. Zhao, S., Liao, J., Li, D., Wang, X. & Li, N. Blending of compatible polymer of intrinsic microporosity (PIM-1) with Tröger's base polymer for gas separation membranes. *J. Membr. Sci.* **566**, 77–86 (2018).
42. Choi, S.-H., Tasselli, F., Jansen, J. C., Barbieri, G. & Drioli, E. Effect of the preparation conditions on the formation of asymmetric poly(vinylidene fluoride) hollow fibre membranes with a dense skin. *Eur. Polym. J.* **46**, 1713–1725 (2010).
43. Ghanem, B. S., McKeown, N. B., Budd, P. M. & Fritsch, D. Polymers of intrinsic microporosity derived from bis(phenazyl) monomers. *Macromolecules* **41**, 1640–1646 (2008).
44. Kosuri, M. R. & Koros, W. J. Defect-free asymmetric hollow fiber membranes from Torlon®, a polyamide–imide polymer, for high-pressure $CO_2$ separations. *J. Membr. Sci.* **320**, 65–72 (2008).
45. Peng, Y. et al. Metal-organic framework nanosheets as building blocks for molecular sieving membranes. *Science* **346**, 1356–1359 (2014).
46. Li, Y. J., Liu, H., Wang, H. T., Qiu, J. S. & Zhang, X. F. GO-guided direct growth of highly oriented metal–organic framework nanosheet membranes for $H_2/CO_2$ separation. *Chem. Sci.* **9**, 4132–4141 (2018).
47. Wang, X. et al. Reversed thermo-switchable molecular sieving membranes composed of two-dimensional metal-organic nanosheets for gas separation. *Nat. Commun.* **8**, 14460 (2017).
48. Peng, Y., Li, Y., Ban, Y. & Yang, W. Two-dimensional metal–organic framework nanosheets for membrane-based gas separation. *Angew. Chem. Int. Ed.* **56**, 9757–9761 (2017).
49. Zhou, S. et al. Development of hydrogen-selective CAU-1 MOF membranes for hydrogen purification by 'dual-metal-source' approach. *Int. J. Hydrogen Energy* **38**, 5338–5347 (2013).
50. Guo, H., Zhu, G., Hewitt, I. J. & Qiu, S. "Twin copper source" growth of metal-organic framework membrane: $Cu_3(BTC)_2$ with high permeability and selectivity for recycling $H_2$. *J. Am. Chem. Soc.* **131**, 1646–1647 (2009).
51. Aguado, S. et al. Facile synthesis of an ultramicroporous MOF tubular membrane with selectivity towards $CO_2$. *New J. Chem.* **35**, 41–44 (2011).
52. Zhang, X. et al. New membrane architecture with high performance: ZIF-8 membrane supported on vertically aligned ZnO nanorods for gas permeation and separation. *Chem. Mater.* **26**, 1975–1981 (2014).
53. Li, Y., Liang, F., Bux, H., Yang, W. & Caro, J. Zeolitic imidazolate framework ZIF-7 based molecular sieve membrane for hydrogen separation. *J. Membr. Sci.* **354**, 48–54 (2010).
54. Yoo, Y., Varela-Guerrero, V. & Jeong, H.-K. Isoreticular metal–organic frameworks and their membranes with enhanced crack resistance and moisture stability by surfactant-assisted drying. *Langmuir* **27**, 2652–2657 (2011).

55. Ranjan, R. & Tsapatsis, M. Microporous metal organic framework membrane on porous support using the seeded growth method. *Chem. Mater.* **21**, 4920–4924 (2009).
56. Shen, J. et al. Subnanometer two-dimensional graphene oxide channels for ultrafast gas sieving. *ACS Nano* **10**, 3398–3409 (2016).
57. Li, X. et al. Efficient $CO_2$ capture by functionalized graphene oxide nanosheets as fillers to fabricate multi-permselective mixed matrix membranes. *ACS Appl. Mater. Interfaces* **7**, 5528–5537 (2015).
58. Kim, H. W. et al. Selective gas transport through few-layered graphene and graphene oxide membranes. *Science* **342**, 91–95 (2013).
59. Li, H. et al. Ultrathin, molecular-sieving graphene oxide membranes for selective hydrogen separation. *Science* **342**, 95–98 (2013).
60. Zhou, F. et al. Ultrathin graphene oxide-based hollow fiber membranes with brush-like CO2-philic agent for highly efficient $CO_2$ capture. *Nat. Commun.* **8**, 2107 (2017).
61. Yang, J. et al. Self-assembly of thiourea-crosslinked graphene oxide framework membranes toward separation of small molecules. *Adv. Mater.* **30**, 1705775 (2018).
62. Cheng, L., Guan, K., Liu, G. & Jin, W. Cysteamine-crosslinked graphene oxide membrane with enhanced hydrogen separation property. *J. Membr. Sci.* **595**, 117568 (2020).
63. Kim, H. W. et al. High-performance $CO_2$-philic graphene oxide membranes under wet-conditions. *Chem. Commun.* **50**, 13563–13566 (2014).
64. Ying, W. et al. Ionic liquid selectively facilitates $CO_2$ transport through graphene oxide membrane. *ACS Nano* **12**, 5385–5393 (2018).
65. Wang, S. et al. A highly permeable graphene oxide membrane with fast and selective transport nanochannels for efficient carbon capture. *Energy Environ. Sci.* **9**, 3107–3112 (2016).
66. Biswal, B. P., Chaudhari, H. D., Banerjee, R. & Kharul, U. K. Chemically stable covalent organic framework (COF)-polybenzimidazole hybrid membranes: enhanced gas separation through pore modulation. *Chem. Eur. J.* **22**, 4695–4699 (2016).
67. Kang, Z. et al. Mixed matrix membranes (MMMs) comprising exfoliated 2D covalent organic frameworks (COFs) for efficient $CO_2$ separation. *Chem. Mater.* **28**, 1277–1285 (2016).
68. Fan, H. et al. Covalent organic framework−covalent organic framework bilayer membranes for highly selective gas separation. *J. Am. Chem. Soc.* **140**, 10094–10098 (2018).
69. Ying, Y. et al. A GO-assisted method for the preparation of ultrathin covalent organic framework membranes for gas separation. *J. Mater. Chem. A* **4**, 13444–13449 (2016).
70. Varoon, K. et al. Dispersible exfoliated zeolite nanosheets and their application as a selective membrane. *Science* **334**, 72–75 (2011).
71. Tang, Z., Dong, J. & Nenoff, T. M. Internal surface modification of MFI-type zeolite membranes for high selectivity and high flux for hydrogen. *Langmuir* **25**, 4848–4852 (2009).
72. Hong, M., Falconer, J. L. & Noble, R. D. Modification of zeolite membranes for $H_2$ separation by catalytic cracking of methyldiethoxysilane. *Ind. Eng. Chem. Res.* **44**, 4035–4041 (2005).
73. Kanezashi, M., O'Brien-Abraham, J., Lin, Y. S. & Suzuki, K. Gas permeation through DDR-type zeolite membranes at high temperatures. *AIChE J.* **54**, 1478–1486 (2008).
74. Kusakabe, K., Yoneshige, S., Murata, A. & Morooka, S. Morphology and gas permeance of ZSM-5-type zeolite membrane formed on a porous c-alumina support tube. *J. Membr. Sci.* **116**, 39–46 (1996).

75. Shekhawat, D., Luebke, D. R. & Pennline, H. W. A review of carbon dioxide selective membranes. *US department of energy* (2003).
76. Kusakabe, K., Kuroda, T., Uchino, K., Hasegawaand, Y. & Mooroka, S. Gas permeation properties of ion-exchanged Faujasite-type zeolite membranes. *AlChE J.* **14**, 1220–1226 (1999).
77. Poshusta, J. C., Tuan, V. A., Pape, E. A., Noble, R. D. & Falconer, J. L. Separation of light gas mixtures using SAPO-34 membranes. *AIChE J.* **46**, 779–789 (2000).
78. Yu, M., Funke, H. H., Noble, R. D. & Falconer, J. L. $H_2$ separation using defect-free, inorganic composite membranes. *J. Am. Chem. Soc.* **133**, 1748–1750 (2011).
79. Wang, D., Wang, Z., Wang, L., Hu, L. & Jin, J. Ultrathin membranes of single-layered $MoS_2$ nanosheets for high-permeance hydrogen separation. *Nanoscale* **7**, 17649–17652 (2015).
80. Shen, Y., Wang, H., Zhang, X. & Zhang, Y. $MoS_2$ nanosheets functionalized composite mixed matrix membrane for enhanced $CO_2$ capture via surface drop-coating method. *ACS Appl. Mater. Interfaces* **8**, 23371–23378 (2016).
81. Achari, A., Sahana, S. & Eswaramoorthy, M. High performance $MoS_2$ membranes: effects of thermally driven phase transition on $CO_2$ separation efficiency. *Energy Environ. Sci.* **9**, 1224–1228 (2016).
82. Chen, D., Ying, W., Guo, Y., Ying, Y. & Peng, X. Enhanced gas separation through nanoconfined ionic liquid in laminated $MoS_2$ membrane. *ACS Appl. Mater. Interfaces* **9**, 44251–44257 (2017).
83. Chen, D. et al. $CO_2$-philic $WS_2$ laminated membranes with a nanoconfined ionic liquid. *J. Mater. Chem. A* **6**, 16566–16573 (2018).
84. Ding, L. et al. MXene molecular sieving membranes for highly efficient gas separation. *Nat. Commun.* **9**, 155 (2018).
85. Shen, J. et al. 2D MXene nanofilms with tunable gas transport channels. *Adv. Funct. Mater.* **28**, 1801511 (2018).
86. Liu, Y., Wang, N., Cao, Z. & Caro, J. Molecular sieving through interlayer galleries. *J. Mater. Chem. A* **2**, 1235–1238 (2014).
87. Liu, Y., Wang, N. & Caro, J. In situ formation of LDH membranes of different microstructures with molecular sieve gas selectivity. *J. Mater. Chem. A* **2**, 5716–5723 (2014).
88. Villalobos, L. F. et al. Large-scale synthesis of crystalline g-$C_3N_4$ nanosheets and high-temperature $H_2$ sieving from assembled films. *Sci. Adv.* **6**, eaay9851 (2020).
89. de Vos, R. M. & Verweij, H. High-selectivity, high-flux silica membranes for gas separation. *Science* **279**, 1710–1711 (1998).
90. Shelekhin, A. B., Dixon, A. G. & Ma, Y. H. Adsorption, permeation, and diffusion of gases in microporous membranes. II. Permeation of gases in microporous glass membranes. *J. Membr. Sci.* **15**, 233–244 (1992).
91. Asaeda, M. & Yamasaki, S. Separation of inorganic/organic gas mixtures by porous silica membranes. *Sep. Purif. Technol.* **25**, 151–159 (2001).
92. Peters, T. A. et al. Hollow fibre microporous silica membranes for gas separation and pervaporation—synthesis, performance and stability. *J. Membr. Sci.* **248**, 73–80 (2005).
93. Elyassi, B., Sahimi, M. & Tsotsis, T. T. Silicon carbide membranes for gas separation applications. *J. Membr. Sci.* **288**, 290–297 (2007).

94. Shiflett, M. B. & Foley, H. C. Ultrasonic deposition of high-selectivity nanoporous carbon membranes. *Science* **285**, 1902–1905 (1999).
95. Jones, C. W. & Koros, W. J. Carbon molecular sieve gas separation membranes-I. Preparation and characterization based on polyimide precursors. *Carbon* **32**, 1419–1425 (1994).
96. Yamamoto, M., Kusakabe, K., Hayashi, J. & Morooka, S. Carbon molecular sieve membrane formed by oxidative carbonization of a copolyimide film coated on a porous support tube. *J. Membr. Sci.* **133**, 195–205 (1997).
97. Centeno, T. A. & Fuertes, A. B. Carbon molecular sieve membranes derived from a phenolic resin supported on porous ceramic tubes. *Sep. Purif. Tech.* **25**, 379–384 (2001).
98. Boutilier, M. S. H. et al. Molecular sieving across centimeter-scale single-layer nanoporous graphene membranes. *ACS Nano* **11**, 5726–5736 (2017).
99. Zhao, J. et al. Etching gas-sieving nanopores in single-layer graphene with an angstrom precision for high-performance gas mixture separation. *Sci. Adv.* **5**, eaav1851 (2019).
100. Celebi, K. et al. Ultimate permeation across atomically thin porous graphene. *Science* **344**, 289–292 (2014).
101. Choi, K., Droudian, A., Wyss, R. M., Schlichting, K.-P. & Park, H. G. Multifunctional wafer-scale graphene membranes for fast ultrafiltration and high permeation gas separation. *Sci. Adv.* **4**, eaau0476 (2018).
102. Huang, S. et al. Single-layer graphene membranes by crack-free transfer for gas mixture separation. *Nat. Commun.* **9**, 2632 (2018).
103. He, G. et al. High-permeance polymer-functionalized single-layer graphene membranes that surpass the postcombustion carbon capture target. *Energy Environ. Sci.* **12**, 3305–3312 (2019).
104. Z. Yuan, et al. Direct chemical vapor deposition synthesis of porous single-layer graphene membranes with high gas permeances and selectivities. *Adv. Mater.* **33**, 2104308 (2021).
105. Robeson, L. M. The upper bound revisited. *J. Membr. Sci.* **320**, 390–400 (2008).
